# HBXIP promotes gastric cancer *via* METTL3-mediated MYC mRNA m6A modification

**DOI:** 10.18632/aging.103767

**Published:** 2020-10-13

**Authors:** Zhi Yang, Xiaodi Jiang, Deming Li, Xiaofeng Jiang

**Affiliations:** 1 Department of General Surgery, The Fourth Affiliated Hospital of China Medical University, Shenyang 110032, P. R. China; 2 Department of Infectious Disease, Shengjing Hospital of China Medical University, Shenyang 110022, P. R. China; 3 Department of Anesthesiology, The Fourth Affiliated Hospital of China Medical University, Shenyang 110032, P. R. China

**Keywords:** gastric cancer, Hepatitis B X-interacting protein, METTL3, N6-methyladenosine methylation, MYC

## Abstract

Gastric cancer (GC) is one of the most common malignancies worldwide with limited treatment options and distinct geographical distribution even in countries such as China. Genetic alterations during its carcinogenesis need urgent elucidation. In this study, we propose an intriguing hypothesis that the hepatitis B X-interacting protein (HBXIP) may function as an oncogene in GC. We harvested 45 GC tissues and matched the paracancerous tissues. The c-myc proto-oncogene (MYC) N6-methyladenosine (m6A) mRNA methylation was detected by m6A RNA immunoprecipitation and dot-blot assays. Expressions of HBXIP, methyltransferase like 3 (METTL3) and MYC were all determined to be upregulated in both GC tissues and cells. Silencing HBXIP led to a decreased expression of METTL3, which inhibited GC cell proliferation, migration and invasion while promoting their apoptosis. Furthermore, METTL3 enhanced MYC m6A methylation and increased MYC translation, which could potentiate the proliferation, migration and invasion of GC cells. Finally, the HBXIP knockdown impeded the tumorigenicity of GC cells *in vivo*. Based on the findings of this study, we conclude that HBXIP plays an oncogenic role in GC *via* METTL3-mediated MYC mRNA m6A modification. The study offers a comprehensive understanding of HBXIP as a potential therapeutic target to limit GC progression.

## INTRODUCTION

Gastric cancer (GC), a prevalent and malignant tumor of the stomach, is the second leading cause of cancer-related mortality not only in China but also worldwide [1, 2]. The incidence rate of GC shows extensive variation depending on region, with the highest rates documented in East Asia and South America but the lowest rates in North America and most parts of Africa [3]. Patients with GC always suffer from preliminary abdominal pain and experience severe symptoms including anorexia, dyspepsia and weight loss along the progression of the disease [1]. Surgical treatment modality such as an endoscopic resection is effective for resolution of the early-stage tumors. However, the tumor cells in advanced GC metastasis to lymph nodes and distant organs, and the recommended treatments of choice for the patients with advanced GC include chemotherapy and other adjuvant therapies such as radiochemotherapy [4]. GC is a multifactorial disease, which can be manipulated by certain environmental and genetic factors. *Helicobacter pylori* (*H. pylori*) have been identified as the chief cause for GC, contributing to about 65 - 80% of new GC cases annually [5]. Genetic factors elicit coordination with the environmental factors for a multifactorial impact and are comprehensively investigated by genome-wide association study (GWAS), which demonstrates MUC1 and PSCA as GC-susceptible genes [6]. Diverse coding genes have been proposed to principally serve as biomarkers in strong association with GC stages and prognosis, including ERBB2, PTEN, PI3K/AKT/mTOR, TP53, HER2 and MYC (also named c-MYC) [7, 8].

Among the biomarkers, MYC fundamentally functions as an oncogene that induces tumor cell growth and inhibits cellular apoptosis in various cancers [9]. For example, MYC modulates numerous microRNAs (miRNAs or miRs) including the oncogenic and antigenic ones essential in modulating liver cancer progression [10]. In the event of GC, MYC upregulates long non-coding RNA (lncRNA) H19 expression to proliferate GC cells and MYC expression represents a prognostic factor in GC patients [11]. METTL3 gene encodes N6-adenosine-methyltransferase that initiates the post-transcriptional methylation of the internal adenosine residues at its N6 site in eukaryotic mRNAs [12]. Research has regarded METTL3 to function as an oncogene that promotes the growth of cancer cells in various cancers such as GC and ovarian carcinoma [13, 14]. More importantly, METTL3 by stimulating the translation process of oncogenes functions as a N6-methyladenosine (m6A) methyltransferase in tumor cells [15]. Additionally, METTL3 can manipulate the methylation of MYC mRNA to facilitate the translation of the MYC protein. In bladder cancer, METTL3 regulates methylation of MYC as well as two specific factors in the nuclear factor-kappaB (NF-κB) pathway to stimulate the overall tumorigenesis [16]. Similarly, METTL3 can increase MYC mRNA level by m6A methylation and stimulate the proliferation of acute myeloid leukemia cells [17]. Hepatitis B X-interacting protein (HBXIP) is a HBV X-interacting protein located in the lysozyme, which is elicited as a contributor to the poor prognosis of GC patients [18]. HBXIP can extensively elevate the expression of METTL3 and concurrently inhibit let-7g so as to promote breast cancer progression [19]. However, the potential involvement of HBXIP in the development of GC is undetermined. Therefore, the current report elucidated the regulation of HBXIP on MYC by employing METTL3, which revealed that methylation of MYC mRNA, increased its stability and subsequently promoted the GC progression.

## RESULTS

### HBXIP was preferentially expressed in GC

**Figure 1:**
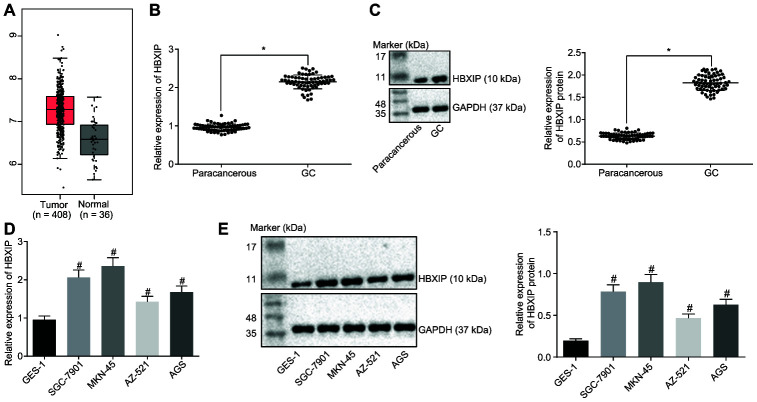
**HBXIP expression pattern is upregulated in GC tissues and cell lines.** (**A**) Expression pattern of HBXIP in tumor tissues and the matched normal tissues analyzed in the TCGA database. The red box on the left represents tumor samples and the gray box on the right represents normal samples. The numbers of tumor samples and normal samples are marked below. (**B**) HBXIP mRNA expression determined by RT-qPCR in GC and paracancerous tissues (n = 45), normalized to GAPDH, * *p* < 0.05 *vs.* the paracancerous tissues. (**C**) Representative Western blots of HBXIP protein and its quantitation in GC and paracancerous tissues (n = 45), normalized to GAPDH, * *p* < 0.05 *vs.* the paracancerous tissues. (**D** and **E**) HBXIP mRNA expression and protein expression patterns in GC cells and normal cells were measured by RT-qPCR (**D**) and Western blot analysis (**E**), normalized to GAPDH, # *p* < 0.05 *vs.* the GES-1 cell lines. The above data were measurement data, and expressed as mean ± standard deviation. Data in panels (**B** and **C**) were compared by paired *t* test and in panels (**D** and **E**) by one-way ANOVA with Tukey’s post hoc test. The cell experiment was repeated 3 times independently.

Firstly, the expression pattern of HBXIP was detected to be upregulated in GC samples by the Cancer Genome Atlas (TCGA) database (Figure 1A). This upregulation was further verified by means of reverse transcription quantitative polymerase chain reaction (RT-qPCR) and Western blot analysis, which demonstrated that the expression of HBXIP in GC tissues was significantly higher compared to that in paracancerous tissues (Figure 1B, 1C). Consistently, the findings revealed a higher expression of HBXIP in the GC cell lines compared to the human normal gastric epithelial cell line GES-1 (Figure 1D, 1E). The aforementioned results signified that HBXIP was highly expressed in both GC tissues and cells.

### Silencing HBXIP inhibited GC cell viability, migration and invasion while inducing apoptosis

**Figure 2:**
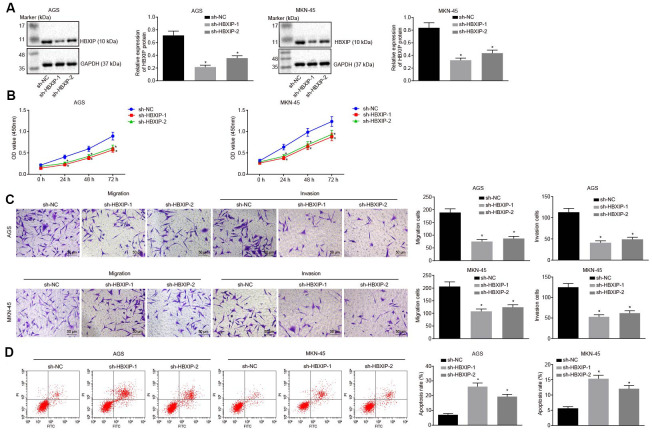
**Silencing HBXIP inhibits GC cell viability, migration and invasion, and induces apoptosis.** (**A**) Representative Western blots of HBXIP protein and its quantitation in AGS and MKN-45 cells upon HBXIP silencing, normalized to GAPDH. (**B**) CCK-8 was used to examine the proliferation of AGS and MKN-45 cells upon HBXIP silencing. (**C**) Transwell assay was used to examine the migration and invasion ability of AGS and MKN-45 cells upon HBXIP silencing (× 200). (**D**) Flow cytometry was used to examine the apoptosis of AGS and MKN-45 cells upon HBXIP silencing. * *p* < 0.05 *vs.* the sh-NC group (AGS or MKN-45 cells treated with sh-NC). The above data were measurement data, and expressed as mean ± standard deviation. Data in panels (**A**, **C** and **D**) were analyzed by one-way ANOVA with Tukey’s post hoc test and in panel B by repeated measures ANOVA with Bonferroni post hoc test. The cell experiment was repeated 3 times independently.

Next, to investigate the impact of HBXIP on the development of GC, the expression of HBXIP was silenced in the GC cells (Figure 2A). The results of cell counting kit-8 (CCK-8) assay (Figure 2B) exhibited a significantly decreased viability of GC cells after silencing HBXIP. Transwell assay (Figure 2C) demonstrated that the migration and invasion abilities of GC cells were attenuated after silencing HBXIP. The results of flow cytometry (Figure 2D) revealed enhanced apoptosis of GC cells upon silencing HBXIP. The preceding findings illustrated that HBXIP knockdown restrained GC progression *in vitro*.

### Silencing HBXIP delayed the progression of GC by downregulating METTL3 expression

TCGA database was employed to analyze the expression pattern of METTL3 and the results revealed it to be increased in the GC samples (Figure 3A). RT-qPCR and Western blot analysis were performed to determine the METTL3 expression pattern in GC tissues and cells, the results of which revealed that the METTL3 expression was elevated in GC tissues and cells in contrast to the paracancerous tissues and normal gastric epithelial cells, respectively, (Figure 3B–3E). The aforementioned data indicated that METTL3 was highly expressed in GC tissues and cells. After silencing HBXIP in the GC cells, the expression of METTL3 in GC cells decreased (Figure 3F). It was indicated that HBXIP silencing downregulated the expression of METTL3 in GC cells.

**Figure 3:**
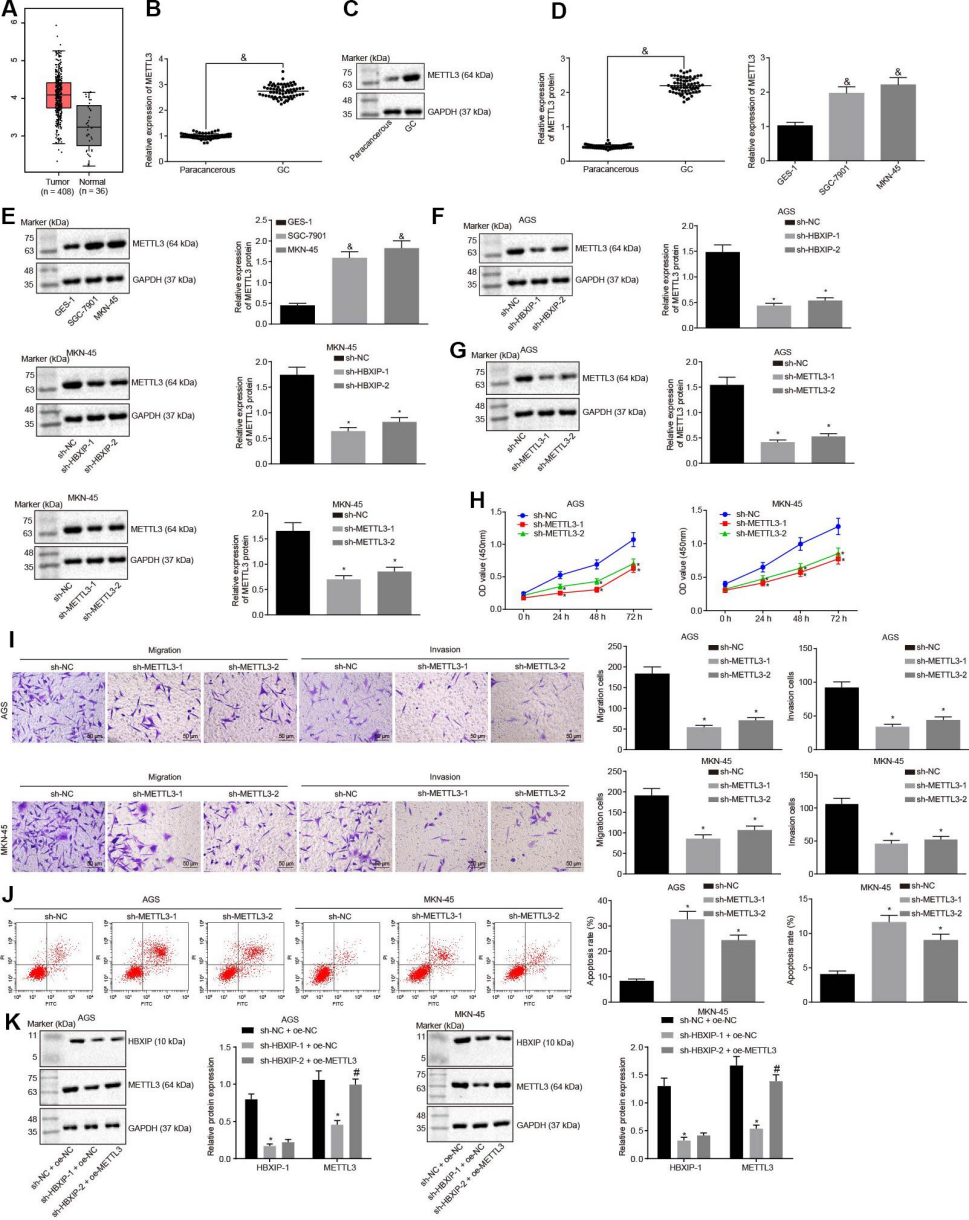
**Silencing HBXIP diminishes the expression pattern of METTL3 and inhibits GC cell viability, migration and invasion, and induces apoptosis.** (**A**) Expression pattern of METTL3 in GC as shown in the TCGA database. The left red box diagram represents tumor samples, and the right gray box diagram indicates normal samples. The numbers of tumor samples and normal samples are marked below. (**B** and **C**), METTL3 mRNA expression and protein expression patterns in GC and paracancerous tissues were determined by RT-qPCR (**B**) and Western blot analysis (**C**), normalized to GAPDH, & *p* < 0.05 *vs.* the paracancerous tissues. **D** and **E**, METTL3 mRNA expression and protein expression in GC cells were determined by RT-qPCR (**D**) and Western blot analysis (**E**), normalized to GAPDH, & *p* < 0.05 *vs.* the GES-1 cells. (**F**) METTL3 protein expression examined by Western blot analysis after silencing HBXIP, normalized to GAPDH. G, METTL3 protein expression examined by Western blot analysis after silencing METTL3, normalized to GAPDH. (**H**) Viability of AGS and MKN-45 cells examined by CCK-8 assay upon METTL3 silencing. (**I**) Migration and invasion of AGS and MKN-45 cells were examined by Transwell assay upon METTL3 silencing (× 200). (**J**) Apoptosis of AGS and MKN-45 cells examined by flow cytometry upon METTL3 silencing. (**K**) Representative Western blots of METTL3 and HBXIP proteins and their quantitation in AGS and MKN-45 cells upon HBXIP silencing or combined with METTL3 overexpression, normalized to GAPDH.

**Figure 3:**
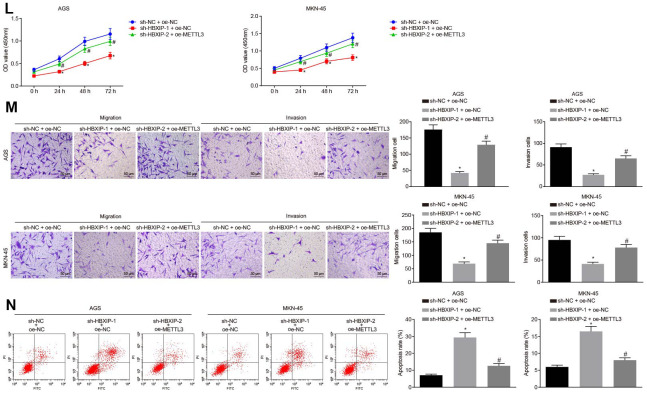
**Silencing HBXIP diminishes the expression pattern of METTL3 and inhibits GC cell viability, migration and invasion, and induces apoptosis.** (**L**) Viability of AGS and MKN-45 cells examined by CCK-8 assay upon HBXIP silencing or combined with METTL3 overexpression. (**M**) Migration and invasion of AGS and MKN-45 cells were examined by Transwell assay upon HBXIP silencing or combined with METTL3 overexpression (× 200). (**N**) Apoptosis of AGS and MKN-45 cells examined by flow cytometry upon HBXIP silencing or combined with METTL3 overexpression. * *p* < 0.05 *vs.* the sh-NC or sh-NC and oe-NC group (AGS or MKN-45 cells treated with sh-NC or both sh-NC and oe-NC). # *p* < 0.05 *vs.* the sh-HBXIP and oe-NC group (AGS or MKN-45 cells treated with both sh-HBXIP and oe-NC). The above results were measurement data, and expressed as mean ± standard deviation. Data in panels **B** and **C** were compared by paired *t* test, in panels (**D**–**G**, **I**–**K**, **M** and **N**) were analyzed by one-way ANOVA with Tukey’s post hoc test, and in panels **H** and **L** by repeated measures ANOVA with Bonferroni post hoc test. The cell experiment was repeated 3 times independently.

To investigate the impact of METTL3 on the biological functions of GC cells, the GC cells were initially infected with lentiviruses expressing short hairpin RNA-negative control (sh-NC), sh-METTL3-1 and sh-METTL3-2. Western blot analysis (Figure 3G) revealed that the expression of METTL3 decreased in GC cells after infection with lentivirus expressing sh-METTL3. The biological functions of GC cells such as proliferation, migration and invasion upon each treatment was then examined. As illustrated in Figure 3H–3J, upon silencing METTL3, the reduced proliferation, migration and invasion ability of GC cells were evident, with acquired apoptosis ability. To further verify whether HBXIP affects the function of GC cells through METTL3, the rescue experiments were designed and performed by delivering lentiviruses expressing sh-NC and overexpression (oe)-NC, sh-HBXIP and oe-NC or sh-HBXIP and oe-METTL3 into the GC cells. Western blot analysis (Figure 3K) findings revealed that the expression of METTL3 in GC cells was lowered by HBXIP silencing, which could be reverted by oe-METTL3. As shown in Figure 3L–3N, the proliferation, migration and invasion ability was suppressed while the apoptosis ability was enhanced in GC cells upon HBXIP silencing, however restoration of METTL3 could partially annul these effects. The aforementioned results demonstrated that overexpression of METTL3 could reverse the inhibitory effect of HBXIP silencing on the proliferation, migration and invasion of GC cells and the promoting effect on apoptosis.

### Silencing METTL3 diminished MYC expression by reducing m6A modification of MYC mRNA in GC cells

**Figure 4:**
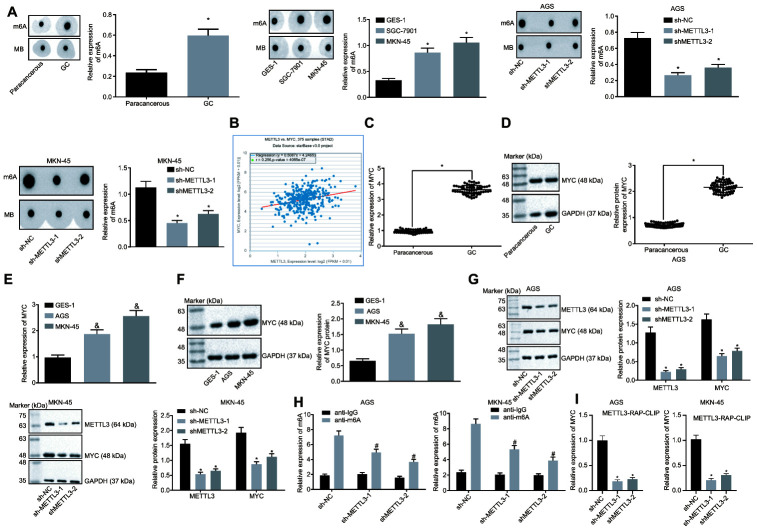
**Silencing of METTL3 reduces MYC mRNA m6A modification and impedes the MYC expression pattern in GC cells.** (**A**) m6A modification levels in GC tissues (n = 45) and GC cells were examined by dot blot assays, * *p* < 0.05 *vs.* the paracancerous tissues or GES-1 cells. (**B**) METTL3 and MYC co-expression in GC from the TCGA database. Each point represents a sample, the abscissa indicates the METTL3 expression pattern, the ordinate indicates the MYC expression pattern, and the upper left indicates the correlation coefficient and *p* value. (**C** and **D**), MYC mRNA expression and protein expression patterns in GC and paracancerous tissues (n = 45) were determined by RT-qPCR (**C**) and Western blot assay (**D**), normalized to GAPDH, which were expressed as mean ± standard deviation, and test with paired *t*-test, * *p* < 0.05 *vs.* the paracancerous tissues. (**E** and **F**) MYC mRNA expression and protein expression patterns in AGS and MKN-45 cells were determined by RT-qPCR (**E**) and Western blot assay (**F**), normalized to GAPDH. (**G**) Representative Western blots of MYC protein and its quantitation in AGS and MKN-45 cells after silencing METTL3, normalized to GAPDH. (**H**) Me-RIP and RT-qPCR were used to examine m6A levels of MYC mRNA in AGS and MKN-45 cells after silencing METTL3. (**I**) The binding of METTL3 to MYC mRNA assessed by PAR-CLIP assay in GC cells following METTL3 silencing. The above results were measurement data, and expressed as mean ± standard deviation. Data in panels **A** (the left), (**C** and **D**) were compared by paired *t* test, and in panels **A** (the middle and the right) and (**E**–**I**) by one-way ANOVA with Tukey’s post hoc test. The cell experiment was repeated 3 times independently.

METTL3 a methylase catalyzes the methylation of m6A in mRNA [20]. The preceding findings demonstrated that METTL3 was highly expressed in GC tissues and cells. To ascertain whether METTL3 affected the m6A modification level of mRNA in GC cells, dot blot assays were performed and the results (Figure 4A) showed that mRNA m6A levels were significantly elevated in GC tissues compared to the paracancerous tissues. In contrast to the human normal gastric epithelial cell line GES-1, the m6A level in GC cells was higher. After silencing METTL3, the m6A level of intracellular mRNA was significantly reduced, suggesting that METTL3 stimulated m6A modification of mRNA in GC cells. In addition, an existing study indicated that the methylation enzyme METTL3 could increase the MYC expression by performing m6A modification of the MYC mRNA in bladder cancer [16]. The co-expression analysis of METTL3 and MYC in GC in the TCGA database (Figure 4B) revealed a positive co-expression relationship between METTL3 and MYC. The findings of Western blot analysis revealed a notably increased expression pattern of MYC in GC tissues and cells (Figure 4C–4F). After silencing METTL3, the MYC expression was significantly reduced in GC cells (Figure 4G), thereby suggesting that METTL3 silencing decreased the expression of MYC in GC cells. The m6A level of MYC mRNA was examined by means of Me-RIP assay, the results of which showed that m6A level of MYC mRNA decreased after silencing METTL3 in GC cells (Figure 4H). Additionally, photo-activatable ribonucleoside-enhanced crosslinking and immunoprecipitation (PAR-CLIP) was performed to identify their relationship, the results of which showed a reduction in the expression of MYC mRNA in the immune complexes upon silencing METTL3 in GC cells (Figure 4I), indicating that the binding of METTL3 to MYC mRNA was weakened following METTL3 silencing. The aforementioned results indicated that the reduction of METTL3 led to decreased MYC expression by attenuating the m6A modification of MYC mRNA in GC cells.

### Silencing of METTL3 hindered proliferation, migration and invasion of GC cells by downregulating m6A modification of MYC mRNA

**Figure 5:**
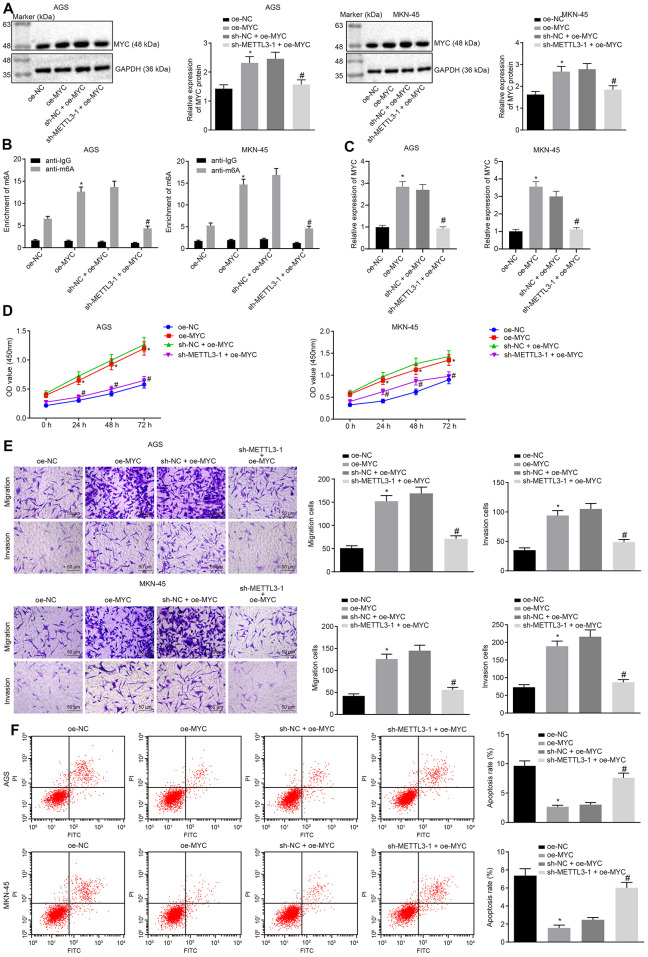
**METTL3 inhibits GC cell viability, migration and invasion, and induces apoptosis by mediating MYC mRNA m6A modification.** (**A**) Representative Western blots of MYC protein and its quantitation in GC cells treated with oe-MYC or combined with sh-METTL3-1, normalized to GAPDH. (**B**) Me-RIP and RT-qPCR were used to examine m6A levels of MYC mRAN in GC cells treated with oe-MYC or combined with sh-METTL3-1. (**C**) The binding of METTL3 to MYC mRNA assessed by PAR-CLIP assay in GC cells treated with oe-MYC or combined with sh-METTL3-1. (**D**) CCK-8 was used to examine the proliferation of GC cells treated with oe-MYC or combined with sh-METTL3-1. (**E**) Transwell was used to examine the migration and invasion of GC cells treated with oe-MYC or combined with sh-METTL3-1 (× 200). (**F**) Flow cytometry was used to examine the apoptosis of GC cells treated with oe-MYC or combined with sh-METTL3-1. * *p* < 0.05 *vs.* the oe-NC group (GC cells treated with oe-NC). # *p* < 0.05 *vs.* the sh-NC + oe-MYC group (GC cells treated with sh-NC and oe-MYC). The above data were all measured data, expressed as mean ± standard deviation. Data in panels (**A**–**C**, **E** and **F**) by one-way ANOVA with Tukey’s post hoc test, and in panel D by repeated measures ANOVA with Bonferroni post hoc test. The cell experiment was repeated 3 times independently.

Next, we investigated the effects of METTL3 on GC through MYC. The cells were infected with the lentivirus expressing oe-MYC or in combination with lentivirus expressing sh-METTL3-1. Data of Western blot analysis (Figure 5A) showed that the MYC expression was increased in cells infected with the lentivirus expressing oe-MYC relative to the cells infected with lentivirus expressing oe-NC. In comparison to the cells infected with the lentivirus expressing both sh-NC and oe-MYC, the MYC expression was markedly reduced in cells infected with lentivirus expressing both sh-METTL3-1 and oe-MYC. Me-RIP assay was adopted to examine the m6A modification level of MYC mRNA in GC cells, the results (Figure 5B) of which showed that the m6A level of MYC mRNA was significantly increased in cells infected with lentivirus expressing oe-MYC compared to the cells infected with lentivirus expressing oe-NC. METTL3 silencing considerably diminished the m6A level of MYC mRNA in the presence of MYC. Besides, the results of PAR-CLIP assay revealed an enhanced binding of METTL3 to MYC mRNA when MYC was overexpressed, which could be reversed by METTL3-1 silencing (Figure 5C). Furthermore, as shown in Figure 5D–5F, compared to the cells infected with lentivirus expressing oe-NC, the proliferation, migration and invasive abilities of GC cells were potentiated in cells infected with the lentivirus expressing oe-MYC, while their apoptosis was decreased. However, METTL3 silencing reversed the simulative effects of MYC overexpression on the proliferation, migration and invasive potentials of GC cells along with the inhibitory effect on the apoptosis ability. The aforementioned results indicated that METTL3 silencing restrained the development of GC by inhibiting m6A modification of MYC mRNA *in vitro*.

### Silencing HBXIP disrupted METTL3-mediated m6A modification of MYC mRNA

**Figure 6:**
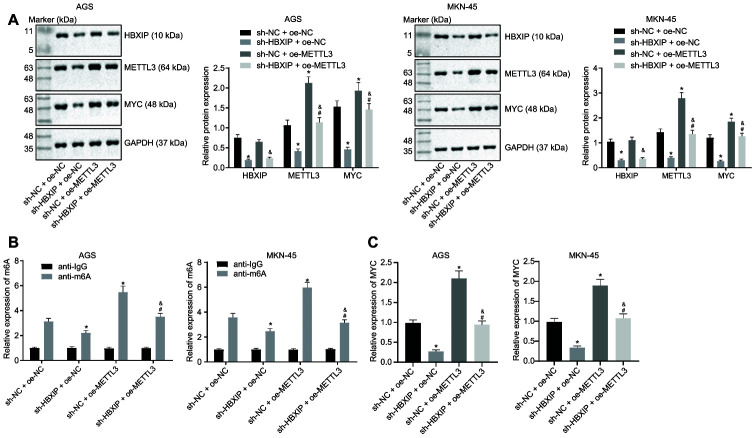
**Silencing HBXIP impairs METTL3-mediated MYC m6A mRNA modification *in vitro*.** (**A**) Representative Western blots of HBXIP, METTL3 and MYC proteins and their quantitation in AGS and MKN-45 cells treated with sh-HBXIP, oe-METTL3 or both, normalized to GAPDH. (**B**) m6A levels of MYC mRNA were examined by Me-RIP and RT-qPCR in AGS and MKN-45 cells treated with sh-HBXIP, oe-METTL3 or both. (**C**) The binding of METTL3 to MYC mRNA assessed by PAR-CLIP assay in GC cells treated with sh-HBXIP, oe-METTL3 or both. * *p* < 0.05 *vs*. the sh-NC and oe-METTL3 group (AGS or MKN-45 cells treated with sh-NC and oe-NC, # *p* < 0.05 *vs.* the sh-HBXIP + oe-NC group (AGS or MKN-45 cells treated with sh-HBXIP and oe-NC), & *p* < 0.05 *vs.* the sh-NC + oe-METTL3 group (AGS or MKN-45 cells treated with sh-NC and oe-METTL3). The above results were measurement data, and expressed as mean ± standard deviation, and compared using one-way ANOVA with Tukey’s post hoc test. The cell experiment was repeated 3 times independently.

On the basis of aforementioned findings, HBXIP could increase the expression of METTL3, and METTL3 could subsequently increase the MYC expression pattern through METTL3-mediated m6A modification. Therefore, we speculated the dependence of METTL3-mediated m6A modification on HBXIP. The results of Western blot analysis (Figure 6A) showed that the expressions of METTL3 and MYC were decreased by HBXIP knockdown while the MYC expression was elevated by restoration of METTL3. Furthermore, the METTL3 gain-of-function could emancipate the expression of MYC inhibited by sh-HBXIP, while the HBXIP loss-of-function diminished the expressions of METTL3 and MYC increased by oe-METTL3. Analyses based on the Me-RIP and PAR-CLIP assays (Figure 6B, 6C) revealed that the m6A level of MYC mRNA and the binding of METTL3 to MYC mRNA were attenuated upon silencing HBXIP, however both of which were enhanced by METTL3 overexpression. Furthermore, restoration of METTL3 annulled the suppression of the m6A level of MYC mRNA and binding of METTL3 to MYC mRNA induced by HBXIP knockdown. These experimental findings signified that HBXIP silencing impaired the m6A modification of MYC mRNA by inhibiting METTL3 *in vitro*.

### Silencing HBXIP repressed the growth of GC *in vivo*

**Figure 7:**
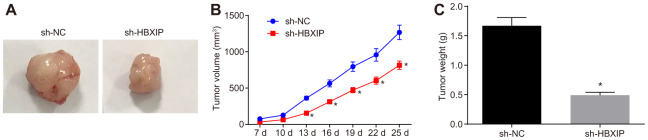
**The growth of GC xenografts is inhibited by silencing HBXIP *in vivo*.** (**A**) Representative images of transplanted tumors in nude mice injected with MKN-45 cells. (**B**) The volume of transplanted tumors monitored every 3 days in nude mice injected with MKN-45 cells. (**C**) Weight of transplanted tumors in nude mice injected with MKN-45 cells. * *p* < 0.05 *vs.* the sh-NC group (nude mice injected with sh-NC-treated MKN-45 cells). Measurement data were expressed as mean ± standard deviation. Data in panel (**C**) were compared by unpaired *t*-test and in panel B by repeated measures ANOVA with Bonferroni post hoc test. n = 6.

Finally, to investigate the impact of HBXIP on the growth of GC xenografts *in vivo*, a tumor xenograft model was established in nude mice. The experimental results (Figure 7A–7C) showed that the tumor volume and weight of mice bearing cells treated with sh-HBXIP decreased significantly, thereby indicating that silencing HBXIP restrained the gastric tumorigenesis.

## DISCUSSION

GC, a malignant tumor of the digestive system is associated with high morbidity and mortality across the globe [21]. Despite advancement in the therapeutics for this malignancy, recent years have only witnessed small increments in the survival outcomes [22]. This raises concern regarding the exploration of the underlying mechanism behind GC development. In the current study, we detected increased expressions of HBXIP, METTL3 and MYC in GC. Contribution of HBXIP to GC development was dependent on m6A modulation on the MYC mRNA mediated by METTL3. Silencing HBXIP could suppress GC cell growth *in vitro* and tumor growth *in vivo*.

A crucial finding of the current study was that the silencing of HBXIP fundamentally reduced METTL3 expression, which consequently impaired the m6A modification of the MYC mRNA. The m6A is a post-transcriptional modulation RNA that regulates MYC mRNA and executes GC progression. Post-transcriptional regulation of RNA contains RNA splicing and RNA modification, which controls the expression level of RNA and more importantly, the delivery of the genetic information [23]. Additionally, m6A is the most prevalent methylation modification in eukaryotic RNA, accounting for over 80% of all RNA methylation. RNA modifications include N1-methyladenosine (m1A), 5-methylcytosine (m5C) and pseudouridine (Ψ) modifications, which are vital during early development and in some human diseases [24]. An existing study demonstrated an association between the aberrant control of m6A homeostasis and various types of cancers including liver cancer, breast cancer, lung cancer and myeloid leukemia. Enrichment of m6A can affect the half-time of RNA, splicing of mRNA, transportation of mRNA out of nucleus and the translation efficiency [25]. Specially, METTL3 induces the methylation of N6-adenosine and can facilitate oncogenic translation by binding to the initiation machinery of translation in human lung cancer [15]. A previous study ascertained METTL3 to manipulate the ARHGAP5 mRNA to stabilize its RNA level in the cytoplasm and finally augment the chemoresistance of GC cells [26]. However, another report elicited that low m6A-indications induce GC cell growth and metastasis by activating Wnt and PI3K-Akt signaling, which is achieved by knockdown of METTL14 instead of METTL3 [27]. Additionally, although the function of HBXIP in GC was ambiguous previously, recent research has shown that HBXIP can promote breast cancer development *via* two mechanisms, one is based on activation of S100A4 expression by binding to its promoter and the other is fundamental to inducing the PTEN/PI3K/AKT signaling pathway [28]. Our findings were consistent with demonstrating the ability of HBXIP to upregulate the expression of METTL3 in order to stimulate the m6A modification of MYC mRNA, we were unable to validate whether HBXIP functioned as a transcriptional factor binding to the promoter region of METTL3, which needed to be studied further.

Another important finding in the current study was that the silencing of HBXIP inhibited GC progression *in vitro* as well as retarded the growth of gastric tumors *in vivo*. An increased positive rate of the HBXIP protein in GC tissues is associated with low overall survival rate of GC patients [18]. In consistency with the preceding finding, a recent study has suggested that the ectopic expression of HBXIP stimulates GC cell proliferation, migration, and invasion while inhibiting apoptosis, which could be induced by positively regulating glucose metabolism. In this metabolism, the regulation of the PI3K/AKT and p53 pathways by HBXIP has been highlighted [29]. However, findings from the current study suggested that silencing HBXIP impeded the progression of GC by inhibiting METTL3. Consistently, overexpression of HBXIP could extensively emancipate the hindered proliferation and enhanced apoptosis driven by METTL3 silencing in breast cancer cells [19]. Moreover, METTL3 was found to be upregulated in GC and was suggested as a major RNA N6-adenosine methyltransferase that could mediate the epithelial-mesenchymal transition signals [30]. An existing study further demonstrated that METTL3 promotes the expression of MYC in bladder cancer cells by augmenting the m6A modification of MYC mRNA [16], which was consistent with our findings. Besides, knockdown of METTL3 can radically impede GC cell proliferation, migration and invasion by targeting the MYC pathway *via* altered m6A modification [31]. MYC was evidently amplified in GC cell lines and tissues, and this amplification is associated with the development and progression of GC [32]. With the findings demonstrating that silencing of METTL3 repressed the development of GC by inhibiting m6A modification of MYC mRNA in GC cells, we attributed HBXIP silencing to induce the blockade of METTL3-mediated m6A modification of MYC mRNA and hence inhibit GC progression.

**Figure 8:**
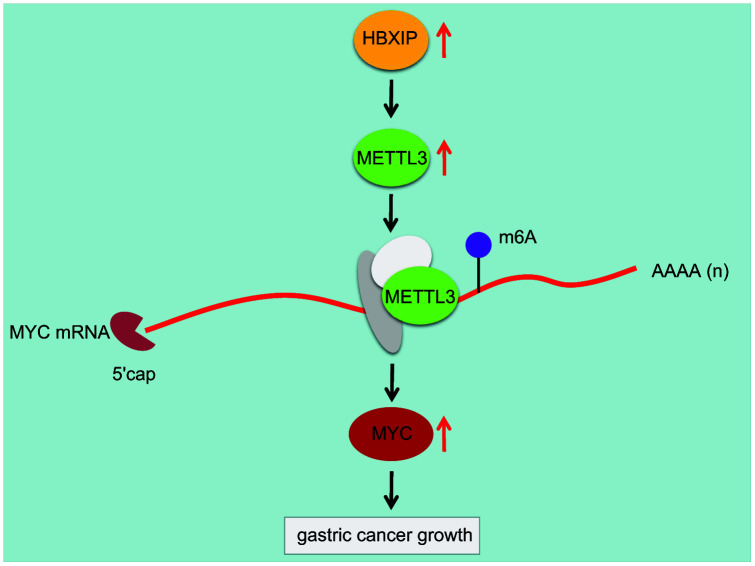
**Mechanism diagram illustrating that HBXIP increased the expression pattern of MYC by mediating METTL3-mediated m6A modification of MYC mRNA, thereby promoting the occurrence and development of GC.**

Overall, we initially observed that the regulatory axis of HBXIP/METTL3/MYC was involved in GC progression (Figure 8). HBXIP could upregulate the expression of METTL3, which successively elevated the m6A modulation of MYC mRNA. Inhibition of HBXIP could reduce the m6A level of MYC mRNA by impairing METTL3-mediated m6A modulation of MYC mRNA, whereby finally hindering the progression of GC in mice. Our findings concluded the functionality of m6A modulation in diverse cancers, which emphasize on the critical function of RNA modulation post-transcriptionally. Even though the role of the HBXIP/METTL3/MYC axis on GC was uncovered by this study, the downstream signaling pathway that regulates the apoptosis of GC cells should be investigated further.

## MATERIALS AND METHODS

### Patient enrollment

In this study, GC tissues and paracancerous tissues were harvested from 45 patients diagnosed with GC (26 males, 19 females, a mean age of 64 years, ranging from 42 to 86 years) who underwent surgery at the Fourth Affiliated Hospital of China Medical University from July 2015 to July 2016. The study was performed with approval of the Ethics Committee of the Fourth Affiliated Hospital of China Medical University, in compliance with the *Declaration of Helsinki*. All participates provided signed informed consent prior to participation.

### Cell culture and lentiviral infection

Four human GC cell lines, SGC-7901, MKN-45, AZ-521, and AGS, as well as a human normal gastric epithelial cell line GES-1 (American Type Culture Collection, Manassas, VA, USA) were cultured in a 5% CO_2_ incubator at 37°C using Roswell Park Memorial Institute 1640 (RPMI-1640) medium containing 10% calf serum, and penicillin-streptomycin solution (1 : 1, final concentration: 100 μ/mL). The cells were detached with 0.25% trypsin, passaged and seeded in a 6-well plate at a density of 3 × 10^5^ cells/well. Upon reaching 70% to 80% cell confluence, the cells were collected for further experimentation.

The lentiviral-based shRNAs targeting HBXIP and METTL3 (sh-HBXIP and sh-METTL3), and lentiviruses overexpressing HBXIP and MYC (oe-HBXIP and oe-METTL3) were generated using the lentiviral vector pSIH1-H1-copGFP (8619936, BioVector Sience Lab Inc., Beijing, China) and lentiviral vector pLV-EGFP-N (VL3211, Inovogen Tech Co., Ltd., Beijing, China), respectively.

The cells were then infected with the lentivirus expressing sh-NC (pSIH1-H1-copGFP-NC), sh-HBXIP (pSIH1-H1-copGFP-HBXIP), sh-METTL3 (pSIH1-H1-copGFP-METTL3), oe-MYC (pLV-EGFP-N-MYC), both sh-NC and oe-METTL3 (pSIH1-H1-copGFP-NC and pLV-EGFP-N-METTL3), sh-HBXIP and oe-NC (pSIH1-H1-copGFP-HBXIP and pLV-EGFP-N-NC), sh-HBXIP and oe-METTL3 (pSIH1-H1-copGFP-HBXIP and pLV-EGFP-N-METTL3), sh-NC and oe-NC (pSIH1-H1-copGFP-NC and pLV-EGFP-N-NC), sh-NC and oe-MYC (pSIH1-H1-copGFP-NC and pLV-EGFP-N-MYC), and sh-METTL3-1 and oe-MYC (pSIH1-H1-copGFP-METTL3 and pLV-EGFP-N-MYC), respectively.

### RT-qPCR

Total RNA content was extracted from tissues and cells using the TRIzol reagents (Invitrogen, USA). The Nanodrop 2000 micro-ultraviolet spectrophotometer (1011U, nanodrop, Molecular Devices, Silicon Valley, CA USA) was used to measure the concentration and purity of the extracted RNA. Next, the extracted RNA content was reversely transcribed into complementary DNA (cDNA) using the PrimeScript RT reagent Kit in strict accordance with the provided instructions (RR047A, Takara Bio, Inc., Shiga, Japan). Primers for HBXIP, METTL3, and MYC were designed and synthesized by TaKaRa Bio, Inc. (Shiga, Japan) (Table 1). Real-time qPCR was performed on an ABI 7500 instrument (Applied Biosystems, Foster City, CA, USA). The relative transcription level of the target gene was calculated based on the relative quantification (2^-ΔΔCT^ method) with glyceraldehyde-3-phosphate dehydrogenase (GAPDH) serving as the internal reference.

**Table 1:** Primer sequences for RT-qPCR.

**Gene**	**Primer sequences**
HBXIP	F: 5'-ATGGAGCCAGGTGCAGGCT-3'
	R: 5'-TGGAGGGATTCTTCATTGTG-3'
METTL3	F: 5'-CAAGCTGCACTTCAGACGAA-3'
	R: 5'-GCTTGGCGTGTGGTCTTT-3'
MYC	F: 5'-CAAGAGGCGAACACACAACGTCT-3'
	R: 5'-AACTGTTCTCGTCGTTTCCGCAA-3'
MYC-m6A	F: 5'-GCATACATCCTGTCCGTCCA-3'
	R: 5'-TGAGCGAAAAAGAGGTTGCTG -3'
GAPDH	F: 5'-GATTCTGCAACTGCAACGCA-3'
	R: 5'-CATGTGGGCCATGAGGTCCACCAC-3'

Note: RT-qPCR, reverse transcription quantitative polymerase chain reaction; HBXIP, Hepatitis B X-interacting protein; METTL3, methyltransferase-like 3; MYC, c-myc proto-oncogene; m6A, N6-methyladenosine; GAPDH, glyceraldehyde-3-phosphate dehydrogenase; F, forward; R, reverse.

### Western blot analysis

Total protein content was extracted from the tissues or cells using radio-immunoprecipitation assay lysis buffer containing phenylmethanesulfonyl fluoride (P0013C, Beyotime Biotechnology Co., Ltd., Shanghai, China). The total protein concentration was measured using a bicinchoninic acid kit. A total of 50 μg protein was dissolved in 2 × sodium dodecyl sulfate (SDS) loading buffer. The protein content was subjected to SDS-polyacrylamide gel electrophoresis for separation, after which the protein content was transferred onto a polyvinylidene fluoride (PVDF) membrane by the wet transfer method. The membrane was then probed with the primary antibodies to HBXIP (ab157480, dilution ratio of 1 : 1000), METTL3 (ab195352, dilution ratio of 1 : 1200), MYC (ab9106, dilution ratio of 1 : 1800), Bcl-2-associated X protein (Bax) (ab32503, dilution ratio of 1 : 2000), B-cell lymphoma-2 (Bcl-2) (ab32124, dilution ratio of 1 : 1000), and GAPDH (ab9485, dilution ratio of 1 : 2500) at 4°C overnight. The PVDF membrane was rinsed 3 times with the Tris-buffered saline with Tween 20 (TBST) for 10 min each time and incubated with the horseradish peroxidase (HRP)-labeled secondary goat anti-rabbit immunoglobulin G (IgG) H&L (HRP) (ab97051, 1 : 2000) for 1 h. The aforementioned antibodies were purchased from Abcam Inc. (Cambridge, MA, USA). Next, the PVDF membrane was rinsed with TBST, placed on clean glass plates and developed using the enhanced chemiluminescence (ECL) reagents (Cat. No. BB-3501, Amersham, Little Chalfont, UK). The membrane was photographed using the Bio-Rad image analysis system (BIO-RAD Laboratories, Hercules, CA, USA), and quantified with the Quantity One v4.6.2 software. The relative protein expression was expressed as the ratio of gray value of the corresponding protein band to that of GAPDH protein band.

### m6A RNA immunoprecipitation

Total RNA content was isolated from the GC cells using the TRIzol reagents, and the mRNA from total RNA content was isolated and purified using the PolyATtract® mRNA Isolation Systems (A-Z5300, A&D Technology Corporation, Beijing, China). Antibody against m6A (1 : 500, ab151230, Abcam Inc., Cambridge, MA, USA) or IgG (ab109489, 1 : 100, Abcam Inc., Cambridge, MA, USA) was supplemented to the IP buffer (20 mM Tris pH 7.5, 140 mM NaCl, 1% NP-40, 2 mM ethylene diamine tetraacetic acid), and then incubated with the protein A/G magnetic beads for 1 h. The isolated and purified mRNA and magnetic bead-antibody complexes were added to the IP buffer containing the ribonuclease inhibitor and protease inhibitor for subsequent incubation at 4°C overnight. The RNA was eluted using the elution buffer and was purified by phenol-chloroform extraction, followed by RT-qPCR analysis. The primer sequences are presented in Table 1.

### PAR-CLIP assay

GC cells were allowed to react with 200 mm 4-thiouridine (Sigma-Aldrich Chemical Company, St Louis, MO, USA) for 14 h and cross-linked with 0.4 J/cm^2^ at 365 nm. After lysis, the specific antibody to METTL3 was supplemented to facilitate immunoprecipitation at 4°C. The precipitated RNA was probed with [g-32-P]-ATP and observed by means of autoradiography, followed by proteinase K digestion. The RNA content was extracted and RT-qPCR was employed to determine the expression pattern of MYC [33].

### Dot blot assays

Total RNA content was isolated from the GC cells using the TRIzol reagents, and the mRNA from the extracted RNA content was isolated and purified using the PolyATtract® mRNA Isolation Systems (A-Z5300, A&D Technology Corporation, Beijing, China). The isolated mRNA was denatured under ultraviolet irradiation for 7 min and frozen on ice. The isolated mRNA was dotted on an Amersham Hybond-N and the membrane was optimized for nucleic acid transfer (GE Healthcare, Boston, MA, USA). After a regimen ultraviolet (UV) cross-linking, the RNA was rinsed with 1 × phosphate buffered saline Tween-20 (PBST) for 5 min after which a membrane blockade was conducted using 5% skim milk powder, followed by incubation with the antibody to m6A at 4°C overnight. Finally, the membrane was visualized using the Immobilon Western Chemilum HRP Substrate (Millipore, Bedford, MA, USA).

### CCK-8 assay

GC cell proliferation was quantified based on the CCK-8 method (CK04, Dojindo, Kumamoto, Japan). Briefly, the GC cells were seeded into 96-well plates at a density of 1 × 10^4^ cells per well and allowed to stand with 10 μL of the CCK-8 reagent for 3-h incubation at 37°C at 0 h, 24 h, 48 h, and 72 h following transfection.

### Transwell assay

Matrigel (YB356234, Shanghai Yubo Biological Technology Co., Ltd., Shanghai, China) stored at -80°C was defrosted and liquefied at 4°C overnight. A total of 200 μL of Matrigel was added to 200 μL of serum-free medium (4°C) and mixed for dilution. A total of 50 μL of Matrigel was added to each apical chamber and incubated in an incubator for 2 - 3 h for solidification. Following cell detachment and counting, the cell suspension was prepared using serum-free medium. A total of 200 μL of the cell suspension was added to the apical chamber of each well, after which 800 μL of the conditioned medium containing 20% fetal bovine serum (FBS) was supplemented to the basolateral chamber. After incubation for 20 - 24 h in the incubator (37°C), the Transwell plate was removed, rinsed twice with phosphate buffer saline (PBS) and immersed in formaldehyde for 10 min. The Transwell plate was stained using 0.1% crystal violet at room temperature for 30 min. The cells on the upper surface were wiped off using a cotton ball. Under an inverted microscope, at least 4 visual fields were randomly selected for cell counting, observation and photographing. Matrigel was not used in Transwell assay for cell migration detection and incubation period was 16 h.

### Annexin-V-fluorescein isothiocyanate/propidium iodide (FITC/PI)-labeled flow cytometry

After 48 h of transfection, the cells were digested with 0.25% trypsin (without ethylenediamine tetraacetic acid) (YB15050057, Shanghai Yubo Biological Technology Co., Ltd., Shanghai, China) and amassed in a flow tube. The supernatant was discarded after centrifugation. The cells were rinsed 3 times with PBS, and then the supernatant was discarded after centrifugation. According to the provided instructions of the Annexin-V-FITC Apoptosis Detection Kit (K201-100, Biovision Inc., CA, USA), the Annexin-V-FITC/PI solution was formulated with Annexin-V-FITC, PI, N-2-hydroxyethylpiperazine-N-ethane-sulphonicacid (HEPES) buffer at a ratio of 1 : 2 : 50. Every 100 μL of the dye solution was added to resuspend 1 × 10^6^ cells, followed by incubation at room temperature for 15 min. Then 1 mL of the HEPES buffer (PB180325, Procell, Wuhan, Hubei, China) was added and mixed by shaking. The FITC and PI fluorescence were examined by activating the band pass filters of 525 nm and 620 nm with a wavelength of 488 nm to examine cell apoptosis.

### Tumor formation in nude mice

Twelve BALB/c male nude mice (age: 6 - 8 weeks, weight: 18 - 22 g) were purchased from SLAC Laboratory Animal Co., Ltd., (Shanghai, China). The GC cell line MKN-45 stably infected with lentivirus expressing sh-HBXIP was prepared into 5 × 10^7^ cells/mL cell suspension. The cell suspension was injected into the left axilla of nude mice using a 1 mL syringe as the sh-HBXIP group (n = 6). The GC cell line MKN-45 infected with the lentivirus expressing sh-NC was dispersed into the cell suspension, which was injected into nude mice as the sh-NC group (n = 6). Tumor growth was observed and data were recorded after inoculation. On the 26^th^ day, all nude mice were euthanized by cervical dislocation and the tumors were resected and weighed. The experimental procedure and animal use were followed with approval of the Animal Ethics Committee of the Fourth Affiliated Hospital of China Medical University.

### Statistical analysis

All experimental data were processed using the SPSS 21.0 software (IBM, Armonk, NY, USA). Measurement data (mean ± standard deviation) between two groups were compared using the paired *t* test and among multiple groups by using one-way analysis of variance (ANOVA), followed by Tukey’s post hoc test. Data at different time points were compared by repeated measures ANOVA, followed by Bonferroni’s post hoc test. The Kaplan-Meier method was adopted to calculate the survival rate of patients, while the Log-rank test was adopted for single factor analysis. In all statistical analysis, a value of *p* < 0.05 was regarded to be significantly different.
